# Fish-on-a-chip: a sensitive detection microfluidic system for alzheimer's disease

**DOI:** 10.1186/1423-0127-18-33

**Published:** 2011-05-28

**Authors:** Jasmine P Devadhasan, Sanghyo Kim, Jeongho An

**Affiliations:** 1 College of Bionanotechnology, Kyungwon University, San 65, Bokjeong-Dong, Sujeong-Gu, Seongnam-Si, Gyeonggi-Do 461-701, Republic of Korea; 2 Department of Polymer Science & Engineering, SungKyunKwan University, Suwon, Gyeonggi-do 440-146, South Korea

**Keywords:** Fluorescence *in situ *hybridization (FISH), Microfluidic chip, Alzheimer's disease (AD), Nanoparticles, Molecular probes

## Abstract

Microfluidics has become an important tool in diagnosing many diseases, including neurological and genetic disorders. Alzheimer's disease (AD) is a neurodegenerative disease that irreversibly and progressively destroys memory, language ability, and thinking skills. Commonly, detection of AD is expensive and complex. Fluorescence in situ hybridization (FISH)-based microfluidic chip platform is capable of diagnosing AD at an early stage and they are effective tools for the diagnosis with low cost, high speed, and high sensitivity. In this review, we tried to provide basic information on the diagnosis of AD via FISH-based microfluidics. Different sample preparations using a microfluidic chip for diagnosis of AD are highlighted. Moreover, rapid innovations in nanotechnology for diagnosis are explained. This review will provide information on dynamic quantification methods for the diagnosis and treatment of AD. The knowledge provided in this review will help develop new integration diagnostic techniques based on FISH and microfluidics.

## Introduction

Fluorescence in situ hybridization (FISH) was developed during the 1980s, for the detection of specific nucleic acid sequences and cytogenetical analysis [1,2]. Further, FISH has replaced conventional methods such as radioisotope probe labeling [3]. Traditional FISH techniques can safely and quantitatively detect many targets, but it is a time-consuming process [4]. Recently, FISH-based microfluidic technique was introduced and was shown to have low cost and high speed. It also offers a number of advantages such as lower amounts of sample and reagents required, less energy, less time required, disposability, compact size, computerization, and trouble-free analysis [5]. Microfluidics is useful for detecting different kinds of samples, such as microorganisms [6], biological materials (DNA, RNA) [7,8], enzymes [9], antibodies [10,11], mammalian cells [12], and biomolecular interactions, and it can also be applied to environmental monitoring, medical diagnostics, the food and agricultural industries [6,13-15], and detection of genetic disorders [5]. Such as the well known genetic diseases are cardiovascular problems, diabetes, cancer, arthritis and Alzheimer's diseases (AD) [16]. Inheritance of AD is complex [17,18] and involves language breakdown, mental confusion, and memory loss [19]. AD was first described by German psychiatrist Alois Alzheimer in 1906 [20]. It is a common and complex disease that has various environmental and genetic aspects [21,22]. One recent report found that 1 in 85 people worldwide will have AD by 2050 [23].

Amyloid precursor protein (APP), presenilin 1 (PS-1), and presenilin 2 (PS-2) and sporadic forms genes such as *apolipoprotein E (APOE) *increase the risk for AD later in life [24]. Therefore, early genetic-based diagnosis is very important for managing AD. FISH-based microfluidic analyses are highly suitable for the detection of single nucleotide polymorphisms (SNP) [5]. Hence, biomarkers and molecular probes are important to detecting AD at an early stage [25]

Alternatively, peptide nucleic acid (PNA) probes can be used for diagnosis instead of DNA probes or as complementary probes to DNA. They exactly mimic DNA probes and therefore one of the most powerful tools for molecular biology and medical diagnostic analysis. PNA can bind to complementary strand of DNA and RNA sequences with high affinity and high specificity [26-28]. PNA-based FISH analyses are used for quantitative telomere analysis using fluorescent-labeled PNA probes [26]. Labeling analysis reveals human telomeric repeat sequences and also can accurately estimate telomere lengths [29]. PNA can also form a triplex with the target double-stranded DNA [30]. Apart from this, duplex invasion, double duplex invasion, and triplex invasion binding are also possible [26]. Perhaps the detection of DNA hybridization by electrochemical method was high compassion, cheaper with advantage of microfabrication technology [31] and using PNA probes for hybridization is equally possible in this technology [32]. Moreover, this PNA shows high affinity with DNA sequences, antisense and antigen agents, biosensors, and molecular probes [30].

This review attempted to summarize the requirements for integrating FISH techniques with microfluidic technology for AD diagnosis. Since, AD detection is possible at an early stage, which is an easy and cheaper detection method. A detailed discussion concerning the detection of AD is carried out.

## FISH-Based Detection of Chromosomal Abnormalities

Though the numbers of automated scanning systems are commercially available, the visual based detection methods are most important for confirmation of results [33,34]. FISH is a great system for identifying chromosomal abnormalities. It can be applied to genetic mapping and diagnosis of novel oncogenes, solid tumors, and various cytogenetic disorders. It also has achieved universal acceptance as a clinical laboratory tool [35,36]. Most importantly, microchip-based FISH techniques can reduce labor time and cost [37]. The human chromosome has been analyzed biochemically and structurally for cytogenetic investigations and diagnostics. FISH technique can also be used to analyze chromosomal details and localize a specific gene, and it is important to develop the fluorescence microscopy [38].

Initially, AD was diagnosed by enzyme-linked immunoassays (ELISA), which is further extended to develop nanoparticle-based bio barcode amplification analysis. Bio barcode assay (BCA) is more than 1 million times more sensitive compared to ELISA, and it uses amyloid β derived diffusible ligands (ADDLs) as a marker. BCA assay can also be used to diagnose AD with about 85% accuracy, due to the high amount of ADDLs present in the cerebrospinal fluid (CSF). However, the drawback of obtaining the CSF sample from the spinal cords [39]. To overcome this difficulty, researchers are trying to design a test that uses blood and urine samples instead.

Recently, Ivan and colleagues reported that human brain diseases such as AD, which are present in the human brain and are associated with chromosomal disorders [40-42]. In this research, chromosome 21 aneuploidy in lymphocytes and fibroblasts cells of AD patients was observed using FISH techniques [43-45]. In this study, three kinds of DNA probes were used, including chromosome enumeration probes [46-48] micro detection probes [49], and five color probes [40,33]. These probes were used along with multicolor FISH techniques. Using this technique, evaluated more than 480,000 neural cells [40,50,51]. FISH analyses on five different chromosomes and 7000 nuclei from seven brain tissue samples were carried out at the same time. The signals were captured using a CCD camera by the quantitative FISH method (cohu, 4910 series, cohu inc., San Deigo, CA) [40]. Several proteins were identified as risk factors of AD, including PS-I, APOE ε4, and amyloid β peptide. According to Takako *et al.*, the PS-I gene is found on chromosome 14q24.3. This single 14q24.3 locus can be detected by FISH [52-54]. Amyloid β peptide is a risk factor for AD and can be found in the urine of AD patients. When analyzed by Western blotting, 0.003 to 1.11 ng in 1 ml of amyloid β peptide was obtained from a urine sample [55]. (Table 1) Summarized the other biomarkers of AD and their sample sources [56-65].

**Table 1:** List of biomarkers for Alzheimer disease and their sample source

Factor	Sample Source	Biomarkers	Ref
Histopathological factors	Urine, Blood	Amyloid beta peptide	[55,56]
	CSF	Tau protein	[57]
	CSF	Phosphorylated tau	[58]

Genetic factor	Blood,saliva	APOE	[59,60]
	CSF, blood,saliva	APP	[61,62,60]
	Blood	PS-1	[63]
	Blood	PS-2	[63]

Synaptic pathological factor	CSF	ADDL	[39]

Others	CSF	Somatostatin	[64]
	Blood	Metal ions	[65]

Other research has proved that FISH is an effective detection technology for AD. Generally, premature centromere separation (PCD) is associated with numerous human diseases [66]. PCD was analyzed using peripheral blood lymphocytes samples of AD patients on chromosome 18. The comparative analysis was carried using elderly samples (as control). FISH has proven that the frequency of PCD is very high on chromosome 18 and that this disease is associated with aneuploidy [67].

As mentioned before, PCD is one of the reasons for causing AD, hence the blood lymphocytes in metaphase stage have used for the cytogenetic analysis. Since individuals with AD contain a high amount of PCD at metaphase stage, disease detection in the micronucleus (MN) of blood lymphocytes can be carried out using FISH. For this reason the chromosome pancentromeric DNA probes has been used [38,68]. FISH technique based results and records have been established to develop this research forth.

FISH is a sensitive technique for detecting cytogenetical disorders, but it also has some drawbacks. FISH analysis requires an efficient and experienced staff as well as a large amount of expensive probes for the experiment (Approximately $90 per slide). Chip-based analysis requires 0.5 to 1 μl of probes, whereas conventional FISH method requires 10 μl of probes. Microfluidic chip-based FISH techniques reduce the cost by 10 to 20-fold [37,38,69,70] as some possible probes impede the high cost effect.

As discussed before, PNA probes used in molecular diagnostic and FISH-based detection [71] can be used to diagnose neurodegenerative dementia, chromosomal disorders such as Parkinson's, frontotemporal dementia (associated with chromosome 17), AD [72], and genomic mutation or labeling of chromosomes [26]. PNA probes are used for *in situ *hybridization to recognize human chromosomes 1, 2, 7, 9, 11, 17, and 18 in metaphase and interphase stage nuclei [73,74]. Multicolor PNA probes are used for the samples such as lymphocytes, aminocytes, and fibroblasts [54,75]. Based on the above literature review, AD at an early stage can be detected on chromosome 18 [67], chromosome 17 [72], and lymphocytes [68] by the FISH method. PNA oligomers can be integrated with a micro total analysis system such as microarray and automatic construction of PNA records array [76,77]. Furthermore, DNA/DNA hybridization, PNA/DNA hybridization, and antigen-antibody interaction for proteins like APP is possible using a microfluidic chip [78].

## Connection of Microfluidic Chip for Detection of Alzheimer's Disease

Highly qualified and efficient technicians spend several hours performing conventional FISH protocol using centromeric probes; it takes a minimum of 2-3 hours to complete the process. However, anyone can manage microfluidic chip-based FISH techniques, as only a few minutes are required to complete this procedure. A FISH-based microfluidic chip device can analyze thousands of genes or thousands of patient samples at a single time, and a technician only needs to spend a few minutes [69]. Many types of analyzing materials are used to study the nervous system [75]. Microfluidic chips are one of the most useful devices for detecting neurodegenerative diseases such as AD. For AD, early identification is important as this type of neurodegenerative disease has dangerous effects in later life [25]. This can be combined with a micro electroporation chip to detect other genetic disorders such as Huntington's disease, autosomal dominant Parkinson's disease, and charcot-marie-tooth disease [79-81]

Moreover, microfluidic chip is one of the most effective tools for disease detection; especially for genetic based diagnoses and other biomedical applications [82-88]. This microfluidic technique has evolved from Micro Electro Mechanical Systems (MEMS). The MEMS eliminated other critical biophysical, chemical and biological analysis [89]. The main aim of this microfluidic system is miniaturization. The major constituent of the microfluidic chip is fabricating materials and controlling the fluid flow [90]. The microfluidic chip accommodates the test fluids and chemicals within the channel to carry out the experiments, where the channel size ranges from 10-100 μm or more in width. Basic principles of microfluidics flow through the channel could be characterized by the Reynolds Number, This is described as Re = ρvL/μ, where L is the most relevant length scale, μ is the fluid viscosity, ρ is the fluid density and v is the average velocity of the flow [91]. There are two common processes for fluid motion: laminar flow and electrokinetic flow. One of the basic laws of the laminar flow is pressure and diffusion to distribution of the molecules transported within the channel. The electrokinetic flow needs electrohydrodynamic force between the inlet and outlet port [92]. The microfluidic device control the movement of fluids via force or electrical energy and integrated optical system finds the solutions of the particular experiment. Different kinds of materials are used to make microfluidic chips and it is useful for different kinds of biochemical analyses, particularly polydimethylsiloxane (PDMS) chips [85,87], paper based chips, and thermoplastic chips, which are very cheap. It also helpful in making inexpensive disposable chip and to avoid the cross contamination of biological samples [93-95]. Recently, advanced techniques incorporated with microfluidic chips were developed for optical, electrical, and mechanical sensing. This can be achieved by connecting or attaching a CCD and CMOS sensor to the microfluidic chip. FISH based chip method shows high sensitivity even with a low amount of sample [96].

Microarray and BCA are major detection tools for biological analysis. Microarrays are a one of the micro total analysis system (μTAS) and it is a direct method for the detection of single nucleotide polymorphisms (SNPs). However, microarrays require an expensive device for analysis and require a long incubation period [97]. The sample should be amplified by polymerase chain reaction (PCR). If the sample concentration is very low, the immobilized sample should be fixed on a glass slide using a microarray spotting machine [98]. It needs a special scanner to analyze the microarray chip. Compared to microarrays, microfluidic chips used for SNP detection require less time and are more sensitive even with a small amount of sample [97]. Ultra high-throughput microfluidic chips are more reliable for disease diagnosis as it focuses on SNP analysis for AD detection and can lead to the development of drugs for SNP. Microfluidic chips have the ability to perform hundreds of reactions and can synthesis up to 10,000 compounds per chip [99].

BCA is a sensitive analytical method for detection of AD and other diseases. It can also be used as a disease-monitoring device and for the analysis of disease markers [39,100]. Moreover, BCA system used to detect amyloid β protein, which is a hallmark of AD [39]. Microfluidic chips have replaced many laboratory tools, including BCA, due to its portability, automation, and simplicity. Hence, BCA continues to be the next stage of development for surface-immobilized BCA [39,100,101], but its working format resembles a microfluidic chip. Integrated microfluidic barcode chips increase the sensitivity of the detection [97,102]. BCA proved the several considerable and new analytical potentialities, though it is not even in its most favorable form. To analyze the target DNA sequence it needs three different kinds of oligonucleotides like magnetic particle capture sequence, universal sequence and barcode capture strand, it is synthetically demanding and expensive. Hybridization of barcode DNA with support strands on Au-NP surface is extremely difficult to attain 100% loading consistently. Skilled person are required for operating this system and should follow several safety procedures when using human samples [103]. Also they have limitation to use the different colors of fluorescent labels in BCA [95,104] and it needs reader like verigene ID to find out the data [47]. But the microfluidic chip has designed with an encapsulated BCA, surface immobilized BCA. This single device is simple, inexpensive, and can be used in many applications such as chemical reactors, sensors, and more [100,102].

**Figure 1:**
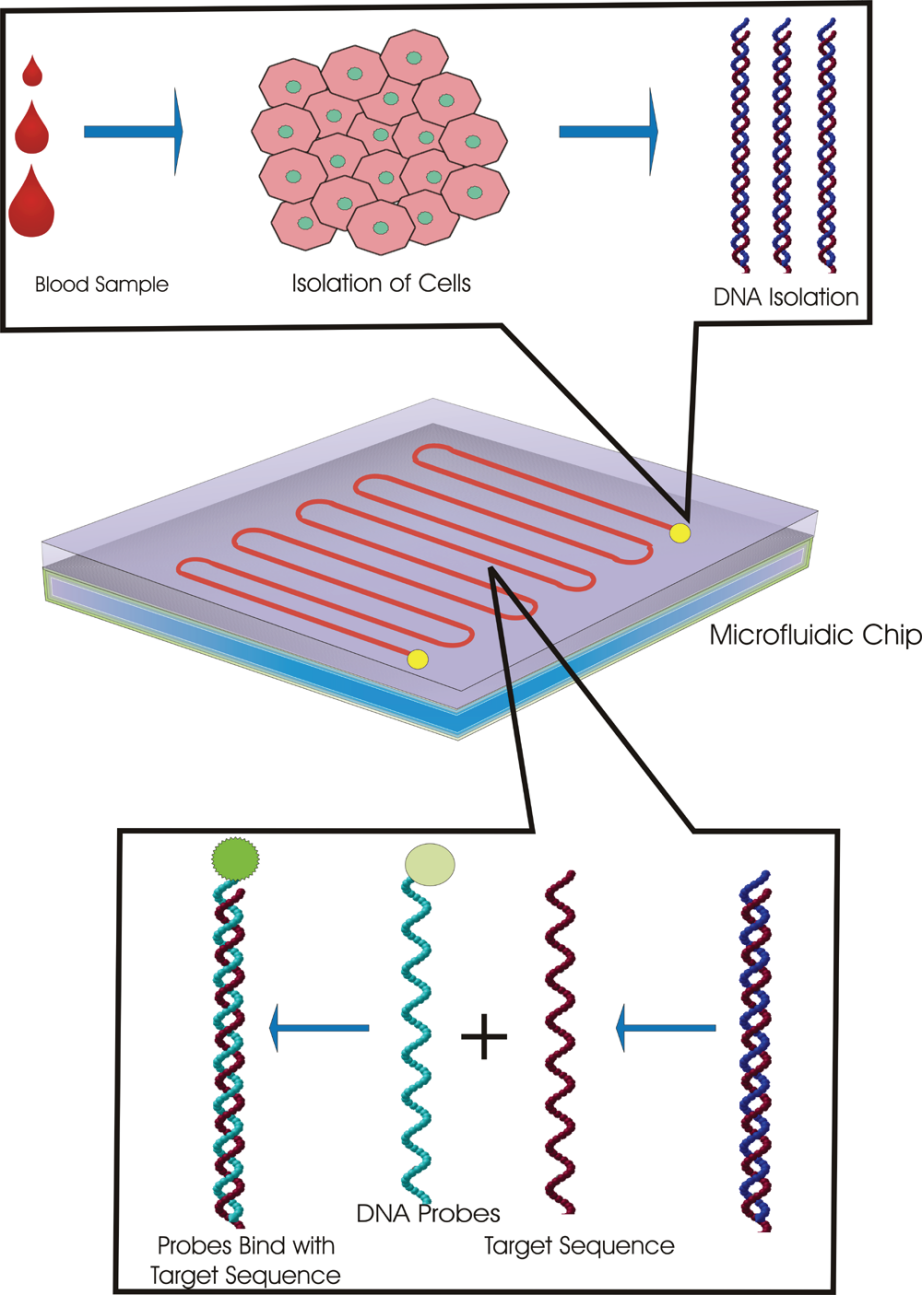
**Analysis of blood sample using FISH based on microfluidic chip**. Blood samples introduced into the microfluidic chip by capillary force and cells were attached on the channel surface by heating the microfluidic chip. Then Proteinase K introduced into the chip to digest the cells, followed by increasing the temperature of microfluidic chip for few sec to denature the DNA, then fluorescence tagged DNA probes introduced into the chip. Complementary DNA probes hybridized with the target sequence and emit fluorescence.

FISH is carried out as an automatic method on a microfluidic chip, as illustrated in Figure 1. Sieben *et al.*, used a microfluidic chip which is useful for distinguishing the chromosomal defects from PBMC cells. The cell suspension were mixed with PBS and introduced in the microchannel by capillary force. This chip should be heated to improve the cell attachment on the channel surface. These cells were treated with proteinase K. The Proteinase K is allowed to digest the cells, and also it is helpful to enter the respective DNA probes with fluorescence into the cells [37]. By using this technique, the detection of chromosomal defects and genetic analysis are possible within 1 hour. This is an important technique to reach the next level in this field. This method is fast, inexpensive and automated genetic screening is applied to distinguish the specific chromosomes defects (aneuploidy) and other related disorders [105]. AD is a major aneuploidy-related disorder. Peripheral blood lymphocytes [40,67] and primary fibroblast cell samples are used for the detection of AD [44]. Since, the microfluidic chip could be accomplished using the raw blood samples, which gives genetic information for many kinds of genetic disorders [99]. This microfluidic chip also can be applied to analysis of urine samples [106], the AD diagnosis could be possibly done by detecting the amyloid β peptide, which is obtained from the urine [55]. Recent types of microfluidic chips reduce the reagent cost by 20-fold and reduce the labor time by 10-fold [99].

## Sample Preparation Method for Microfluidic Chip-Based Diagnosis of Alzheimer's Disease

**Figure 2:**
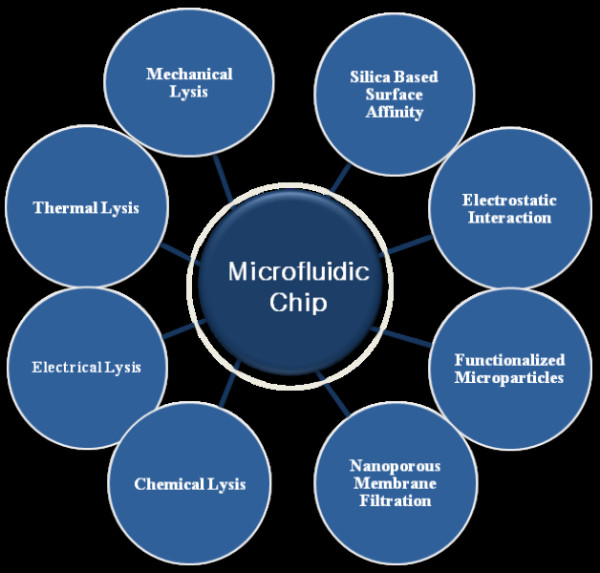
**Sample preparation using microfluidic system: Cell lysis and Nucleic acid extraction method**. Cell lysis is rupture of cell membrane and release of cell components by using cell lysis methods such as a) Mechanical lysis b) Thermal lysis c) Chemical lysis d) Electrical lysis. Nucleic acid extraction is the isolation of DNA and RNA from the cells using microfluidic chip by a)Silica-based surface affinity b) Electrostatic interaction c) Nanoporous membrane filtration d) Functionalized microparticles

Sample preparation plays a major role in microfluidic chip-based analysis. Easy sample preparation helps reduce the difficulties of analysis in a short period of time. Chip-based analysis is an authentic technique like PCR, capillary electrophoresis, FISH [103], and other technologies that use nano wires [107] and nano pores [108]. In conventional methods, nucleic acid sample preparation has high labor cost and requires many steps to isolate nucleic acids from raw materials like blood, spinal fluid, saliva, and tissue. This method takes a long time to prepare the sample for biological analysis [109-111]. However, microfluidic chip-based systems avoid the difficulties of sample preparation, as it is a simple method with low cost and reagent consumption [112]. Microfluidic chip-based systems also do not require any spinning method. Chip-based sample preparation miniaturizes the entire laboratory tool in a single device with micro fabrication method and it replaces the costly equipment used for biological laboratory and clinical field [112]. This lab on chip sample preparation leads to the development of home-based self-analysis. The sample preparation includes two major steps: 1) cell lysis and 2) DNA, RNA extraction, as shown in Figure 2 Cell lysis is classified into four major types: 1) mechanical lysis 2) thermal lysis 3) chemical lysis, and 4) electrical lysis [112]. Lysis techniques aim to rupture the cell wall and release cell cytoplasm. For mechanical lysis, the cell membranes disturbed with mechanical force such as microknives [112,113], poly methylsiloxane (PDMS) membrane [114], ultra sonication [115,116], and laser beam irradiation have been used [117] to release the cytoplasm. APP can be obtained by rupturing the single membrane of CSF cells. APP has about 590-680 amino acids present in the cytoplasmic tail [61]. The mismetabolism of APP increases the risk of AD. Hence, measuring the amount of APP in CSF would help detect AD [118]. Although, APP is traditionally measured by Western blotting [119], however the blotting technique does not produce accurate measurements. Therefore, it is not a truly quantitative method for APP measurement in CSF [118].

Thermal lysis is a pertinent technique for DNA and RNA isolation and involves heating the sample at 100°C for 40 sec in boiling water. The technique is enough to obtain nucleic acids without any damage [120,121]. Microheating is an advanced technique that can be integrated within a microfluidic chip to isolate cell samples from blood and other sources [122]. As we discussed in Section 1, obtaining sample from the CSF is very difficult and painful, although very accurate. To avoid this problem, researchers have developed, blood sample-based methods for detection of AD using FISH techniques and microfluidic chips. Blood lymphocytes used for AD detection along with FISH have shown positive results **[**67]. Since AD is associated with chromosome 21 of human blood, the soluble form of APP can be isolated from human platelets. This isolated APP can be confirmed by immunological methods and Western blotting techniques [62].

Chemical lysis is the other important method for microfluidic-based sample preparation [112]. This can be carried out using buffers and lytic agents such as ammonium chloride [119], SDS, lysozyme, chaotropic salts, β-mercaptoethanol, and Triton-X4 that maintain protein structure and function [123-126]. Amyloid β peptide is normally released from cells that contain small 42 residue proteins fragments. Any person with a decreased amount of amyloid β peptides in CSF would be prone to develop AD [56], which has been confirmed by many studies [127]. The electrical lysis method requires electrical force to lyse the cell membrane. High intensity pulsed electric fields [PEFs] are suitable techniques for microfluidic applications, and it increases the frequency in the μTAS analysis system [128,129]. The advantage of an electrical lysis system with an integrated microfluidic chip is reduced electrical power consumption; specifically 8.5 V at 10 kHz AC is enough to lyse mammalian cells with competence of 74% at low power [130]. From the above discussion, it is clear that any lysis method can be integrated into a microfluidic chip.

From the existing literature, it can be clearly understood that detection at the molecular level surely has an impact on AD diagnosis. Microfluidic chip-based DNA separation can give positive results. As we reported, microfluidic-based DNA separation can be classified into four types: 1) silica-based surface affinity, 2) electrostatic interaction, 3) nanoporous membrane filtration, and 4) functionalized microparticles [112].

Increased surface affinity and high ionic solutions are helpful in binding DNA to glass fiber and silica, due to low electrostatic repulsion and wash out the DNA with low ionic strength buffer. This is the common procedure for DNA extraction. Chaotropic salt solutions have confirmed the results of DNA adsorption and desorption with nanogram quantities of silica resins in microfabricated devices, showing ~70% binding capacity in white blood cells [131]. On the other hand, hybrid architectures of microfabricated devices of silica beads and sol-gel matrix produce ~90% DNA extraction within 15 min [132,133]. However, the drawbacks of hybrid architecture are bonding and shrinkage, which affect the DNA extraction quantity and purity of the sample. Developing tetramethylorthosilicate [TMOS]-based sol-gel matrices with micro pore tools could avoid this problem, thus offering promising potential by extracting DNA from human CSF and viral DNA [134,135]. *APOE *(chromosome 19) from human blood leukocytes cells can also be used for AD detection. Usually, the conventional method for isolation of the *APOE *gene takes a long time, approximately 6-8 hours, and also requires gene amplification [59]. On the other hand, microfluidic-based DNA preparation and identification is faster and requires a smaller amount of sample. Further, AD patients contain a high amount of tau-protein in their CSF [57]. Conventional methods measure tau protein by ELISA [136], which can measure 10 pg/ml of tau-protein in CSF. Using microfluidic chip-based techniques, tau protein can be measured in combination with high throughput analysis methods such as surface plamon resonance (SPR) [137].

*APOE *is a glycoprotein containing 299 amino acids and with a molecular weight of 34.2 kDa [138]. Usually, *APOE *genes are used as biomarkers for individuals suspected of having AD [52]. *APOE *is classified into three major forms, ApoE2, ApoE3, and ApoE4, the allelic forms of which are *e2, e3*, and *e4*, respectively [139]. Generally, an individual containing the *e2 *allelic form is not at risk for AD. However, an individual containing the *e3 *or *e4 *allelic form is at higher risk to AD at early stage [52].

Integrated silicon-based microfluidic chips are useful for cell lysis and DNA extraction from whole blood [140], and they are very easy to handle [141]. Another method for DNA extraction is based on electrostatic interactions. Chitosan-coated microfluidic chips are often used for DNA extraction from whole blood and cell lysis at pH levels near 5, with a rate of isolation of 68% for human genomic DNA [142]. Functionalized microparticles and magnetic beads are used for sample preparation with the aid of an integrated microfluidic chip [112]. Genetic diseases can be detected by obtaining DNA from cells in the saliva [143]. The risk for AD of APP and amyloid β proteins isolated from saliva was analyzed by ELISA [60]. Functionalized magnetic beads were used for saliva sample preparation with the help of lysis buffer. This method could isolate and purify DNA within 10 min [143]. After sample preparation, the microfluidic chip can be used to identify the results.

## Nanomaterials Coupled With Fluorescent Probes for Diagnosis of Alzheimer'S Disease

A pertinent technique is being developed for the optical detection of molecular disease [144]. Organic and inorganic-based nanomaterials exhibit immense potential and have excellent physicochemical visual magnetic properties, which can be easily manipulated [145-147]. Gold nanoparticles, quantum dots, and magnetic nanoparticles are used for diagnosis. Among these, gold nanoparticles play a major role in sensing applications and can be used as a multiprobe tool for visual inspection, fluorescence, Raman scattering, atomic and magnetic force, and electrical conductivity. *In situ *hybridization with nanoparticles can provide sensitive results. FISH coupled with nanoparticles has provided remarkable detection in both prokaryotic [148] eukaryotic cells [149]. A microfabricated device coupled with nanoparticles is 100,000 times more specific and 10 times more sensitive for DNA detection than modern genomic detection systems. The targeted disease sequence DNA presented in the sample on the chip could be bound with gold nanoparticle. Further, additional use of silver solution in the microfluidic chip can produce accurate detection with a minute amount of DNA [144]. In nanoparticle-based DNA detection, a multiple number of DNA probes could bind and detect millions of DNA sequences simultaneously. Further, it leads to the sensor based detection for the different types of biological materials [144,150].

In molecular diagnosis, the nanoparticles are used as a nanoscale material due to their low toxicity, easy pairing with biomolecules, and their adaptability in various detection methods for analyses. Nanoparticle-based microfluidic analysis for biomolecules can produce the data by Fluorescence Raman Scattering or Optical Absorption. The low sample volumes used in microfluidic chips allow the device to concentrate and amplify signals from gold particles [151]. Biotin-labeled PNA probes immobilized on the gold surface could bind with the DNA target sequence with high affinity [152].

Nanoparticles are used in many microscale diagnostic devices such as microarrays and BCA. A microfluidic chip with nanoparticles is used for recognizing specific DNA sequences and can be confirmed by fluorescence detection. Gold nanoparticles are incorporated into the channel wall of the microfluidic chip. The DNA probes are then linked to the monolayer through thiol groups at one end and the fluorescence dye at the other end. Hybridization and detection of target genes can be carried out via *in situ *hybridization on the microfluidic channel. Therefore, this is a promising method for clinical diagnosis [153].

Silver nanoparticles are also useful for DNA detection applications even at a low sample concentration. An integrated PDMS microfluidic chip with high sensitivity when coupled with nanoparticles allows detection in confocal and non-confocal modes [98]. Nanoparticle-based detection of DNA hybridization and protein binding can be carried out by label-based and label-free methods. Label-based detection uses fluorescent nanoparticle labels. On the other hand, label-free detection uses sensor, SPR, surface enhance Raman scattering, and cantilever-based biosensor to detect microfluidic data in the laboratory. However, quantitative and sensitive detection is required for taking these methods to the next level of disease treatment [154]. Localized SPR of nanoparticles can be used to detect tau-protein in CSF even at picogram quantities [137]. Further, CMOS sensors used for DNA hybridization detection have been reported in micro fabricated devices [155]. These self-analytical tools are cheap, sensitive, and user-friendly. They can also be incorporated into cell phones, digital cameras, and scanners to enumerate the results and documentation [156,157].

## Future Perspectives

There are many methods and devices used to detect AD, including microarray technology, BCA, microfluidic platform, and antigen antibody-based detection. All of these techniques can be used for biological and clinical analysis. Prevention or early detection is the best solution for avoiding cytogenetic and other dangerous disorders. Patients might be unaware until harmful symptoms are felt. Usually, AD detection is a costly procedure. Hence we tried to develop a cost-effective, microchip-based AD and genetic-based diagnostic techniques for early diagnosis. Self-analyses are available for the detection of flu-like illnesses and colds. A physician can treat patients based on the data obtained from self-analysis. In the future, detecting AD could be made easier using FISH integrated with a microfluidic chip. This detection system would be possible at any stage of the disease. Genetic analysis is the most effective method for biological analysis and disease diagnosis using tissue, body fluids, and microbial analysis. DNA-based detection is a promising method for detecting the suppressed stage of genes. This detection method can be improved as a self-analyzing tool by using a single chip. Above all, this will be a cheaper method and the detection will be molecular level in every clinical visit. This kind of diagnosis will help reduce the number of individuals with AD in the future. This integrated microfluidic chip will be used widely not only for AD treatment but also for all neurobiological diseases. Moreover, the sample collection will not be a challenging task, and detection can be possibly carried out by using blood samples and urine.

## Summary

This review discussed the stages and causes of AD. Conventional detection methods of AD have been using biomolecules obtained from CSF. The detection of AD using FISH techniques and microfluidic chip technology has been carried out in an appropriate manner. Detailed DNA isolation starting from cell lysis was also discussed. The role of nanomaterials in the detection of genetic disease using DNA was reviewed as well. This review envisioned FISH techniques incorporated with microfluidic devices as an innovative diagnostic tool. This method would be helpful for genetic level analysis and early stage analysis of diseases. This single chip will be useful for multiple analyses of DNA hybridization, protein analysis, and other quantitative-based analysis.

As revealed by FISH, microfluidic chip and nanoparticle-based analyses are very effective for diagnosing AD. Our aim is to join all of these effective techniques together. An integrated system would achieve biotherapy of genetic diseases. This work has increased our understanding of AD using microfluidic chips. These fusion techniques will be a common and sensitive tool for all the biological techniques.

## List of Abbreviations

AD: Alzheimer's disease; ADDL: Amyloid β derived diffusible ligands; APOE: Apolipoprotein E; APP: Amyloid precursor protein; BCA: Bio barcode assay; CSF: Cerebral spinal fluid; ELISA: Enzyme-linked immune sorbent assays; FISH: Fluorescence *in situ *hybridization; PCD: Premature centromere division; PNA: Peptide nucleic acid; PS-1: Presenilin 1; PS-2: Presenilin 2; SNP: Single nucleotide polymorphisms; SPR: Surface plasmon resonance

### Authors Contributions

JPD performed the FISH- based detection for chromosomal abnormalities and microfluidic chip- based detection for Alzheimer's Disease. SK designed the work and contributed in Sample preparation method for microfluidic chip- based diagnosis of Alzheimer's disease. JA participated in Nanomaterials coupled with fluorescent probes for diagnosis of Alzheimer's disease and performed for future perspectives.   
